# Trajectories of immune-related serum proteins and quality of life in patients with pancreatic and other periampullary cancer: the CHAMP study

**DOI:** 10.1186/s12885-023-11562-2

**Published:** 2023-11-07

**Authors:** Sofie Olsson Hau, Maja Svensson, Alexandra Petersson, Jakob Eberhard, Karin Jirström

**Affiliations:** Division of Oncology and Therapeutic Pathology, Department of Clinical Sciences, Lund University, Lund, Sweden

**Keywords:** Pancreatic cancer, Periampullary cancer, Health related quality of life, Immune-associated serum proteins, Routine biomarkers, CD28, Decorin, GZMB, GZMH, MCP-1

## Abstract

**Background:**

There is still a profound lack of efficient therapeutic strategies against pancreatic and other periampullary adenocarcinoma. Surgery is seldom possible, leaving palliative chemotherapy the only option for most patients. Chemotherapy treatment is however often accompanied by serious side-effects, and the identification of biomarkers for early prediction of disease and treatment-associated symptoms could help alleviate patient suffering. This study investigated the dynamic interrelationship between immune-related serum proteins, routine biomarkers, and health-related quality of life (HRQoL) factors during chemotherapy treatment of patients enrolled in the prospective, observational study Chemotherapy, Host response And Molecular dynamics in Periampullary cancer (CHAMP).

**Methods:**

Proximity extension assay was applied to analyse 92 immune-associated proteins in longitudinal serum samples from 75 patients, 18 treated with curative and 57 with palliative intent. HRQoL data were available from all patients at baseline (BL), from 41 patients at three months, and from 23 patients at six months. Information on routine laboratory parameters albumin, CA19-9, CEA and CRP were collected from medical charts.

**Results:**

In total nine proteins; chemokine (C–C motif) ligand 23 (CCL23), cluster of differentiation 4 (CD4), cluster of differentiation 28 (CD28), decorin (DCN), galectin-1 (Gal-1), granzyme B (GZMB), granzyme H (GZMH), matrix metallopeptidase 7 (MMP7), and monocyte chemotactic protein-1 (MCP-1) were strongly correlated (Spearman’s Rho ≤ -0.6 or ≥ 0.6) with either cognitive functioning (DCN), emotional functioning (DCN, MCP-1), dyspnoea (CD28, GZMB, GZMH) or insomnia (CCL23, CD4, Gal-1, MMP7) during treatment. Associations between routine laboratory parameters (CA 19–9, CA-125, CRP, CEA and albumin) and HRQoL factors were overall weaker. None of the investigated proteins were associated with pain.

**Conclusions:**

This is, to our knowledge, the first study exploring associations between serum biomarkers and HRQoL in patients with pancreatic or other periampullary cancer, and some findings merit further validation. The associations of DCN and MCP-1with impaired cognitive and/or emotional functioning are of particular interest, given their established link to various neurodegenerative conditions. Chemotherapy is known to cause persistent cognitive dysfunction with effects on memory and executive function, referred to as “chemo brain”. It would therefore be of great value to identify biomarkers for early detection and management of this debilitating condition.

**Trial registration:**

Clinical Trial Registration: NCT03724994.

**Supplementary Information:**

The online version contains supplementary material available at 10.1186/s12885-023-11562-2.

## Introduction

Pancreatic cancer is a grievous disease with a 5-year survival rate of approximately 10%. It is currently the third leading cancer-related cause of death worldwide and projected to rise to the second by 2030 [1, 2]. Because of the diffuse symptomatology, pancreatic cancer is often diagnosed at a late stage in which most tumours are locally advanced or have metastasised. Hence, for the majority of patients, palliative chemotherapy is the only treatment option left. Most tumours are located in the pancreatic head, and the precise anatomic origin of an unresected tumour can be difficult to distinguish from other, less common, tumours arising in the periampullary area, i.e., the distal bile duct or the Ampulla of Vater. These tumours are therefore treated with similar chemotherapy regimens, whereas tumours arising in the periampullary duodenum are treated with different regimens. Approximately 15–20% of patients with pancreatic or other periampullary cancer are eligible for surgery with curative intent followed by adjuvant triple chemotherapy with fluorouracil, oxaliplatin and irinotecan (FOLFIRINOX) for 6 months if tolerated, and double or single agent chemotherapy is given to frailer patients [3, 4]. Some patients have borderline resectable disease and receive chemotherapy preoperatively. Gemcitabine is given as a single agent to patients with co-morbidities or with decreased performance status, or together with *nab*-paclitaxel to patients with intermediate performance status, both in the adjuvant and in the palliative setting. Although treatment with FOLFIRINOX has increased overall survival (OS), toxicity is often severe, with up to 68% of patients suffering from grade 3–4 adverse events such as neutropenia, thrombocytopenia, fatigue, vomiting, diarrhoea, and sensory neuropathy [5–7]. Apart from experiencing toxicity to treatment, patients often suffer from symptoms such as pain, fatigue, and cachexia. In a systematic review by Kristensen et al. in 2016 [8], chemotherapy treatment was shown to be associated with increased health related quality of life (HRQoL) in some studies [9, 10], and with decreased HRQoL in some studies [11–13], but the majority of studies reported stable HRQoL-scores over time. However, although chemotherapy treatment does improve HRQoL in some patients, the benefit is still often outweighed by adverse side effects, since most tumours develop resistance to treatment already in an early stage of the disease. Adding to this, the intrinsic aggressiveness of the disease leads to a rapid deterioration in physical and psychological well-being of the afflicted patients. Therefore, personalised treatment strategies should not only focus on modulating the disease, but also the accompanying illness.

Inflammation plays a crucial role in carcinogenesis, with pro-inflammatory cytokines being secreted by tumour cells and adjacent normal tissue, thus attracting leucocytes and inducing an inflammatory tumour microenvironment [14]. Pro-inflammatory cytokines also induce fever, cachexia, and fatigue [15–17]. Cancer-related fatigue affects more than half of cancer patients and has a major impact on their lives. It is regarded by the patients as being more important than either pain or nausea [18]. In a quantitative review from 2007, a positive association between cancer-related fatigue and circulating levels of interleukin-6 (IL-6), interleukin-1ra and neopterin was found [19]. Furthermore, patients undergoing treatment also suffer from chemotherapy-induced fatigue and it has been suggested that this may well be due to a treatment-induced increase of pro-inflammatory cytokines such as IL-6, IL-8 and IL-10 [20–22].

To the best of our knowledge, no studies have yet investigated HRQoL in relation to levels of inflammatory serum proteins in patients with pancreatic or other periampullary cancer during chemotherapy treatment. The aim of this study was therefore to identify putative biomarkers for improved management and early prevention of disease and treatment-related symptoms in these patients, by analysing real-world data on how trajectories of circulating immune-associated serum proteins align with HRQoL before start of chemotherapy, and after 3 and 6 months, respectively.

## Methods

### Patients

The Chemotherapy, Host response And Molecular dynamics in Periampullary cancer (CHAMP) study is an ongoing prospective, single-arm observational study (Clinicaltrials.gov identifier NCT03724994) [23, 24]. All patients with a diagnosis of pancreatic or other periampullary adenocarcinoma undergoing neoadjuvant, adjuvant or palliative first line chemotherapy treatment at the Department of Oncology, Skåne University Hospital, are invited to participate in the study. Exclusion criteria are patients having another concomitant life-threatening disease and patients who are unable to receive chemotherapy. Clinical and pathology data are compiled at study entry with follow-up being performed at three-month intervals. Serial blood sampling is performed before each chemotherapy treatment cycle and at the end of treatment. HRQoL is assessed every three months through EORTC-QLQ-C30 (The European Organisation for Research and Treatment of Cancer Quality of Life Questionnaire) [25].

### Serum samples and proximity extension assay

Serum samples from baseline (BL), one month, three months and end of treatment (EOT) were obtained from whole blood samples after centrifugation at 2000 g for 10 min and stored in -80 °C until analysis. The serum samples were thawed, randomized, and pipetted onto 96-well plates, refrozen to -80 °C and analysed by Olink Proteomics, Uppsala, Sweden. For the present study, the Olink Immuno-Oncology panel investigating 92 proteins was chosen based on previous literature linking inflammatory proteins with HRQoL factors. In brief, the proximity extension assay uses paired antibody-oligonucleotide-conjugates which bind to specific target proteins [26]. Upon binding, the oligonucleotides hybridize and form a surrogate DNA marker for the specific protein, which is amplified using a quantitative polymerase chain reaction (qPCR) and measured using real-time PCR. The protein concentrations are presented on an arbitrary log_2_ scale called normalized protein expression (NPX) [27].

### Routine laboratory biomarkers

Information on routine laboratory parameters were collected from patient charts. At BL, levels of carbohydrate antigen 19–9 (CA19-9) and albumin were measured in all 75 patients, carcinoembryonic antigen (CEA) was measured in 71 patients and C-reactive protein (CRP) in 72 patients. At three months CA19-9 was measured in 28 patients, CEA in 18 patients, CRP in 28 patients and albumin in 34 patients. End of treatment levels of CA19-9 were measured in 63 patients, CEA in 50 patients, CRP in 51 patients and albumin in 68 patients.

### The EORTC QLQ-C30 questionnaire

The questionnaire comprises of 30 questions and is divided into functional and symptom scales. The functional scales question physical, emotional, role, cognitive and social functioning as well as global health status (5, 4, 2, 2, 2, 2 questions, respectively). A high functional level is indicated by a high score in these scales. Contrastingly, a high score on symptom scales indicates an increased severity of symptoms. Three symptom scales (for nausea and vomiting, fatigue and pain) comprise of two questions each, the remaining six symptoms (dyspnoea, insomnia, appetite loss, constipation, diarrhoea, financial difficulties) are single questions and assess various physical symptoms as well as financial impact. Before statistical analysis, the raw EORTC QLQ-C30 scores were linearly transformed to a 0–100 scale [28]. A mean change of 10 to 20 points at different time points was defined as a moderate change in HRQoL and a change of more than 20 points corresponded to a large change, a change of > 10 was defined as the minimal clinically important difference (MCID) in accordance with Osoba et al. [29].

### Statistical analysis

Statistical analyses were conducted in R version 4.2.2 [30], RStudio version 2022.02.3 + 492 [31] and SPSS® version 27.0.1.0 (IBM SPSS Statistics for Windows, Armonk, NY: IBM Corp). Spearman correlation coefficients between serum proteins and HRQoL factors were calculated using the function *cor* from the R package *stats* and visualised with the function *corrplot* from the package *corrplot *[32]. IL-1 alpha, IL13, IL2, IL33 and IL5 were excluded from this analysis because they contained more than 50% of NPX values below the Limit of Detection (LoD). Coefficients greater than or equal to 0.6 or smaller than or equal to -0.6 were considered strong correlations. Bootstrapped confidence intervals and p-values for the Spearman correlation coefficients were calculated using *ci_cor* (package *confintr *[33]) and *cor.test* (package *stats*) respectively. The ‘bca’ (bias-corrected accelerated) bootstrap method was applied.

Changes in protein levels over time were investigated using the function *olink_ttest* from the R package *OlinkAnalyze* which performs a Welch 2-sample t-test and corrects for multiple testing using the Benjamini–Hochberg method [34]. Linear regression was fit to each protein and overall survival (OS) at each timepoint using the function *lm* from the *stats* package in R. The plots were produced using the R package *ggplot2* and *forestplot *[35, 36]*.* Univariable and multivariable Cox regression was calculated using the function *coxph* from the R package *survival* and applied for analysis of OS in relation to the investigated parameters, using continuous scores for serum proteins and HRQoL scores and log_2_ transformed values for routine parameters [37]. The multivariable analyses included adjustment for performance status and treatment intention, and sex was also included in the analyses related to HRQoL factors, given the previously shown differences between women and men [24]. The significance level was set to 0.1% for the Cox regression analysis.

## Results

### Patient demography

**Fig. 1:**
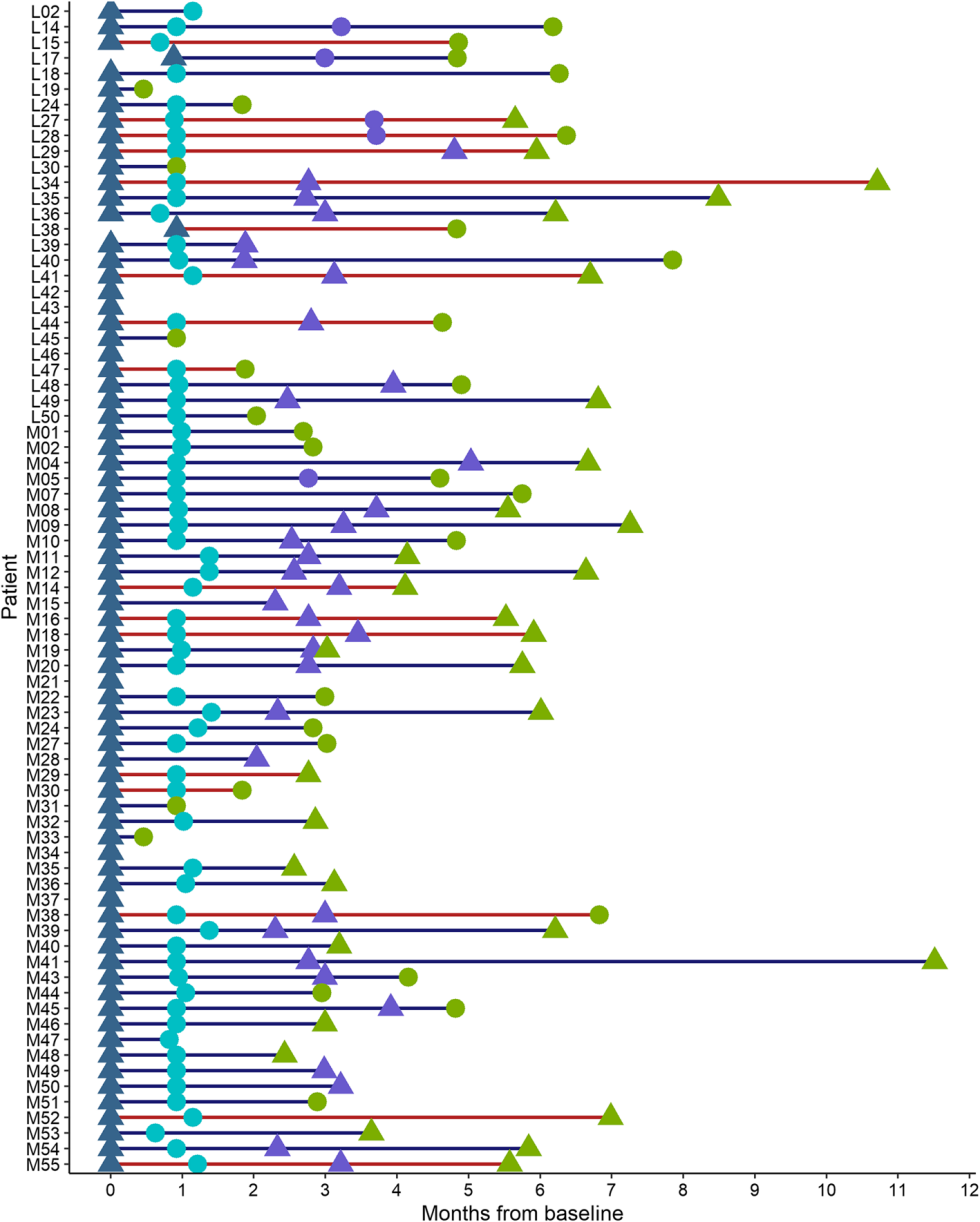
Overview of patients and timepoints for completed HRQoL questionnaires and serum samples. Triangle = timepoint for HR-QoL and serum sample, circle = timepoint for serum sample. Dark blue = baseline, bright blue = one month, purple = three month and green = end of treatment. Blue line = palliative treatment, red line = adjuvant treatment

This study includes 75 patients with completed EORCT-QLQ-30 questionnaires at baseline, as previously described [24]. Of these patients, 41 had completed questionnaires at three months and 23 at six months. The vast majority (73/75) of the patients had a tumour deemed to be of pancreatic origin, according to the pathology and/or radiology reports. In two cases, the resected tumour originated in the ampulla of Vater and distal bile duct, respectively, according to the pathology report, and both were classified as being of pancreatobiliary morphology. All patients treated with curative intent (*n* = 18) had their tumours resected prior to receiving adjuvant chemotherapy. Patient and tumour characteristics have been detailed in a previous study [24]. Among adjuvant treated patients, 8(44%) received gemcitabine-capecitabine, 5(28%) received gemcitabine, 1(6%) received nab-paclitaxel, and 4(22%) received oxaliplatin. Among palliative patients, 9(16%) received gemcitabine, 26(46%) received nab-paclitaxel, and 22(38%) received oxaliplatin. At last follow-up on April 30, 2023, 18 patients were alive, 12 of whom were treated with adjuvant intent and 6 with palliative intent. Median follow-up was 21.5 (range 3.6–51.4) months for patients receiving adjuvant treatment and 7.5 (range 1.4- 50.4) months for patients receiving palliative treatment. An overview of all patients and timepoints of completed questionnaires and serum samples are shown in Fig. 1.

### Levels of exploratory and routine laboratory biomarkers at different time points

**Fig. 2:**
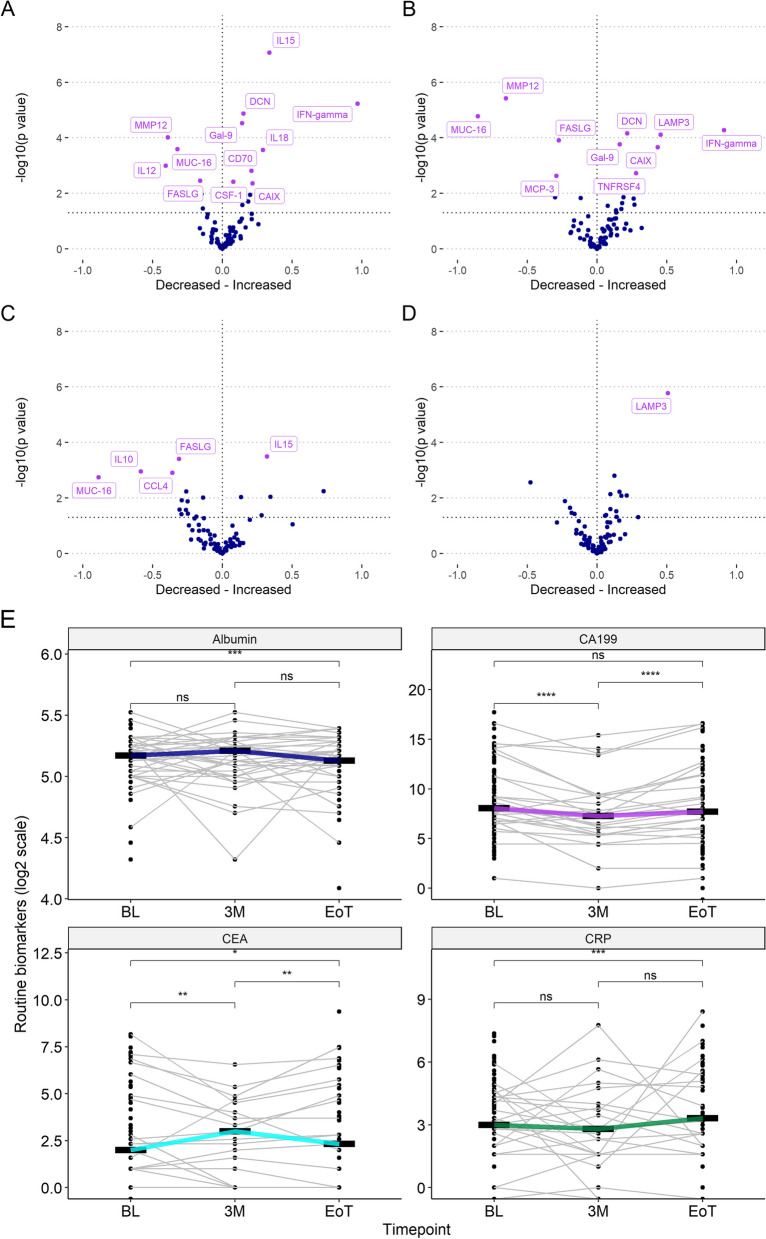
Deregulated serum proteins during treatment. Volcano plots of deregulated serum proteins between **a**) baseline and one month, **b**) baseline and three months, **c**) baseline and end of treatment (six months) and **d**) one month to three months. **e**) Levels of routine biomarkers in log_2_ scale. Bars indicate median value

Top deregulated exploratory proteins are shown in Fig. 2. When adjusted for multiple testing, the top up-regulated protein from baseline to one month was interferon-γ (IFN-γ), followed by interleukin-15 (IL-15), interleukin-18 (IL-18), colony stimulating factor 1 (CSF-1), carbonic anhydrase IX (CAIX), cluster of differentiation 70 (CD70), decorin (DCN) and galectin-9 (Gal-9). The top down-regulated protein was interleukin-12 (IL-12), followed by matrix metalloprotease 12 (MMP12), mucin-16 (MUC-16) and fas ligand (FASLG) (Fig. 2a). The top up-regulated protein at three months compared to baseline was IFN-γ, followed by lysosome-associated membrane glycoprotein 3 (LAMP3), CAIX, tumour necrosis factor receptor superfamily member 4 (TNFRSF4), DCN and Gal-9. The top down-regulated protein at three months compared to baseline was MUC-16, followed by MMP12, monocyte chemotactic protein-3 (MCP-3) and FASLG (Fig. 2b). Il-15 was up-regulated at EOT compared to baseline. The top down-regulated protein at EOT compared to baseline was MUC-16, followed interleukin-10, (IL-10), chemokine (C–C motif) ligand 4 (CCL4) and FASLG (Fig. 2c). LAMP3 was up regulated at three months compared to one month, no other proteins were de-regulated at this timepoint (Fig. 2d).

Trends in levels of the routine biomarkers’ albumin, carbohydrate antigen 19–9 (CA19-9), carcinoembryonic antigen (CEA) and C-reactive protein (CRP) are shown in Fig. 2e. Albumin levels were significantly lower at EOT compared to baseline. CA-19–9 levels were significantly lower at three months compared to baseline but significantly higher at EOT compared to three months. CEA levels were significantly higher at three months and EOT compared to baseline and significantly lower at EOT compared to baseline. CRP levels were higher at EOT compared to baseline.

### HRQoL scores at different time points

Mean and median HRQoL scores and MCID at baseline, three months and EOT are shown in Table 1. A statistically significant increased global health (*p* = 0.013), increased emotional functioning (*p* = 0.010), decreased nausea and vomiting (*p* = 0.006), decreased pain (*p* = 0.006), decreased dyspnoea (*p* = 0.011), improved appetite (*p* = 0.009) and less constipation (*p* = 0.002) was seen from baseline to three months, and a statistically significant increased appetite was seen from baseline to EOT (*p* = 0.005). There were no significant differences between HRQoL at EOT and three months. When examining changes according to MCID a large improvement was seen in appetite at three months and EOT (score 22 and 27, respectively) compared to baseline. Moderate improvements in pain (score 20 and 13, respectively) and constipation (score 15 and 13, respectively) were seen at three months and EOT compared to baseline. A Moderate improvement in global health, as well as less insomnia was seen at three months compared to baseline (score 12 and 12, respectively).

**Table 1:** Changes in health-related quality of life over time

	**BL – 3 M**	**MCID**	***p-value***	**BL—EOT**	**MCID**	***p-value***	**3 M—EOT**	**MCID**	***p-value***
N	75–41			75–23			41–23		
**Global health status** Mean, median score	52–64, 50–67,	**12**	***0.029***	52–62, 50–67,	10	*0.078*	64–62, 67–67	2	*0.407*
**Physical functioning** Mean, median score	71–75, 73–80	4	*0.705*	70–75, 73–80	5	*0.739*	75–75, 80–80	0	*0.053*
**Role functioning** Mean, median score	57–62, 50–67	5	*0.644*	57–61, 50–67	4	*0.508*	62–61, 67–67	1	*0.686*
**Emotional functioning** Mean, median score	67–77, 67–83,	10	***0.002***	67–74, 67–83	7	*0.124*	77–74, 83–83	3	*0.813*
**Cognitive functioning** Mean, median score	82–83, 83–83	1	*0.876*	82–78, 83–83	4	*0.276*	83–78, 83–83	5	*0.334*
**Social functioning** Mean, median score	61–67, 67–67	6	*0.329*	61–67, 67–67	6	*0.196*	67–67, 67–67	0	*0.407*
**Fatigue** Mean, median score	50–46, 44–33	4	*0.960*	50–44, 44–33	6	*0.917*	46–44, 33–33	2	*0.964*
**Nausea and vomiting** Mean, median score	14–7, 17–0	7	***0.002***	14–9, 17–0	5	*0.377*	7–9, 0–0	2	*0.257*
**Pain** Mean, median score	40–20, 33–17,	**20**	*0.206*	40–28, 33–17,	**13**	*0.622*	20–28, 17–17	8	*0.185*
**Dyspnoea** Mean, median score	23–33, 33–33,	10	***0.039***	23–23, 33–0	0	*0.782*	33–23, 33–0,	10	*0.356*
**Insomnia** Mean, median score	31–19, 33–0,	**12**	*0.196*	31–26, 33–0,	5	*0.420*	19–26, 0–0	7	*0.608*
**Appetite loss** Mean, median score	47–25, 33–0,	**22**	***0.001***	47–20, 33–0,	**27**	***0.001***	25–20, 0–0	5	*1.000*
**Constipation** Mean, median score	25–10, 33–0,	**15**	***0.003***	25–13, 33–0	**13**	*0.206*	10–13, 0–0	3	*0.317*
**Diarrhoea** Mean, median score	21–21, 0–0	0	*0.674*	21–22, 0–0	1	*0.157*	21–22, 0–0	1	*0.317*
**Financial difficulties** Mean, median score	8–8, 0–0	0	*0.660*	8–8, 0–0	0	*0.157*	8–8, 0–0	0	*0.157*

*BL* Baseline, *EOT* End of Treatment, *MCID* minimal clinically important difference, with scores of 10–20 indicating a moderate change and > 20 indicating a large change in HRQoL. Non-parametric Spearman’s rank test applied for continuous variables. For functional scores, a high score indicates a high functional level, for symptom scores a high value indicates an increased severity of symptoms

### Proteins associated with HRQoL

Spearman’s correlation was performed for all exploratory proteins and symptoms at all timepoints (Additional file 1). Since patients reported overall low scores for nausea and vomiting, constipation, diarrhoea, and financial difficulties, and the correlations between these factors and investigated proteins were overall weak and these parameters were not included in the subsequent analyses.

**Fig. 3:**
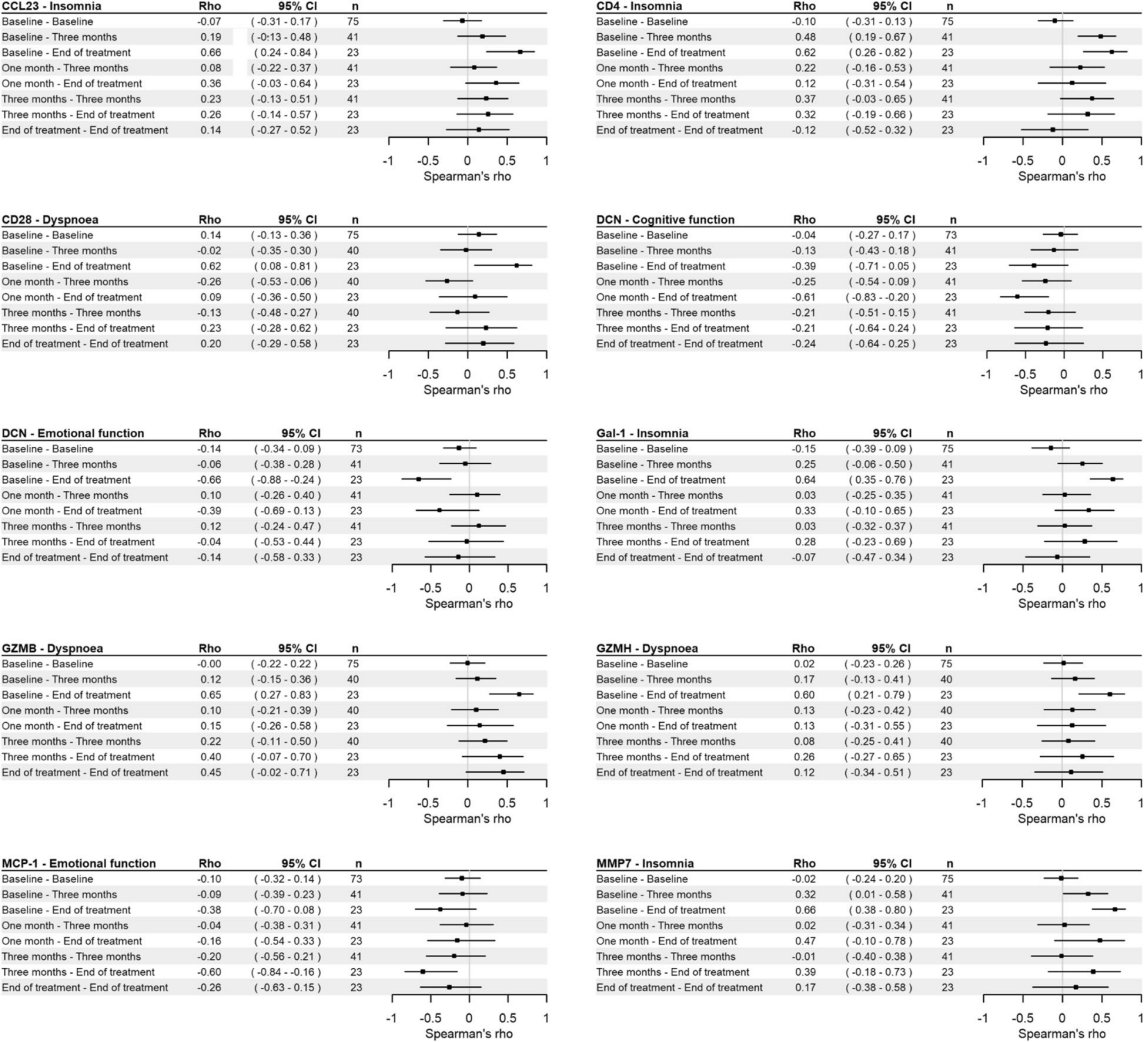
Correlations of serum proteins with HRQoL factors. Spearman’s rho with 95% confidence interval was calculated at all timepoints for proteins with a significant correlation to a HRQoL factor at any timepoint

Proteins with a strong correlation (Spearman’s correlation coefficient ≥ 0.6) to any symptom at any timepoint; chemokine (C–C motif) ligand 23 (CCL23), cluster of differentiation 4 (CD4), cluster of differentiation 28 **(**CD28), decorin (DCN), galectin-1 (Gal-1), granzyme B (GZMB), granzyme H (GZMH), matrix metallopeptidase 7 (MMP7), and monocyte chemotactic protein-1 (MCP-1), were selected for further analysis at all timepoints, and correlation coefficients are visualized in Fig. 3.

Higher levels of CCL23, CD4, Gal-1 and MMP7 at baseline were all strongly associated with insomnia at EOT. There were also moderate correlations between CD4 at baseline and CD4 at three months, respectively, and insomnia at three months. Higher levels of CD28, GZMB and GZMH at baseline were all strongly associated with dyspnoea at EOT. There was also a moderate correlation between GZMB at EOT and insomnia at EOT. High levels of DCN at BL and MCP-1 at three months were strongly associated with decreased emotional function at EOT. High levels of DCN at one month were also strongly associated with decreased cognitive function at EOT.

Given their positive correlations between cognitive and/or emotional functioning we also examined whether levels of DCN and MCP-1 were associated with patient age and sex. There was a significant correlation between age and increased DCN levels at EOT (R = 0.62, *p* = 0.002), but not at any other timepoint, and DCN levels did not differ between sexes. MCP-1 levels did not correlate with either sex or age.

There were no strong correlations between any routine laboratory parameters with HRQoL factor s at any time point except between CEA at three months and decreased appetite at EOT (Additional file 2).

### Prognostic value of investigative factors, routine laboratory parameters and health-related quality of life factors

**Fig. 4:**
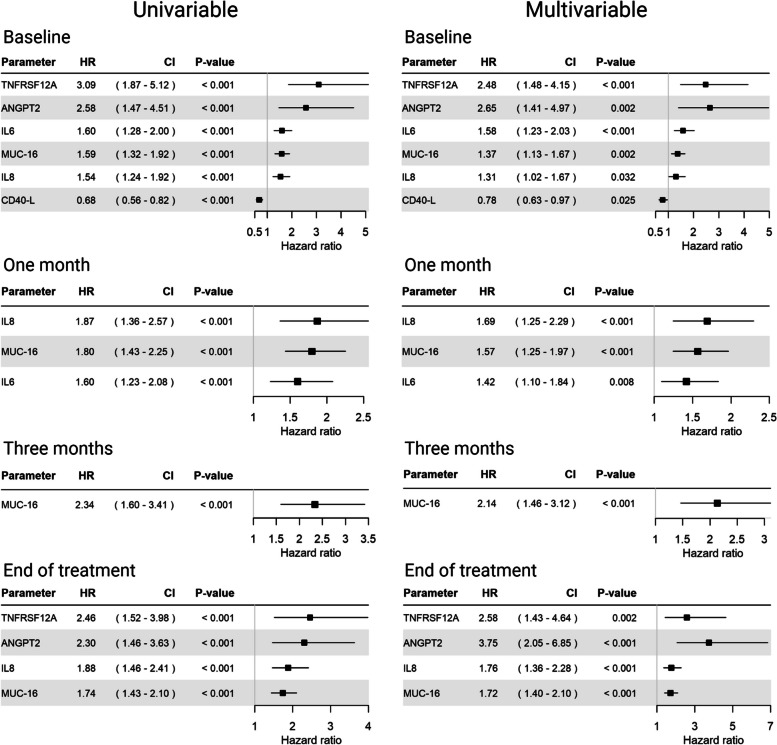
Cox regression analysis of overall survival in relation to levels of serum proteins. Hazard ratios with 99.9% confidence intervals for death at all time points in univariable and multivariable analysis, adjusted for treatment intention and performance status at baseline

Linear regression was applied to select the top prognostic proteins (p-value ≤ 0.001) at each timepoint, which were then further examined with Cox regression analysis. It total, six proteins fulfilled the criteria: TNF Receptor Superfamily Member 12A (TNFRSF12A), Angiopoietin-2 (ANGPT2), interleukin 6 (IL-6), mucin-16 (MUC-16), interleukin 8 (IL-8), and cluster of differentiation 40 ligand (CD40L). Unadjusted and adjusted (for treatment intention and performance status) hazard ratios for death in relation to the most significant proteins at different timepoints are shown in Fig. 4. High levels of all proteins except CD40L were associated with a shorter OS in both unadjusted and adjusted analysis. CD40L was only prognostic at baseline, with high levels being associated with a longer OS in both unadjusted and adjusted analysis. MUC-16, also known as cancer antigen 125 (CA125), was the only protein that was prognostic at all timepoints, and none of the other proteins were prognostic at three months.

Unadjusted and adjusted (for treatment intention and performance status) hazard ratios for death in relation to routine parameters are shown in Additional file 3. None of these parameters were prognostic at three months. High levels of CEA and CA19-9 were associated with a shorter OS in both univariable and multivariable analysis at baseline and EOT. CRP was only prognostic at baseline, with high levels being associated with a shorter OS in both unadjusted and adjusted analysis. In contrast, albumin was only prognostic at EOT, with high levels being associated with a longer OS in both unadjusted and adjusted analysis.

Unadjusted and adjusted hazard ratios for death in relation to HRQoL factors at different timepoints are shown in Additional file 4. High physical functioning at baseline and three months was significantly associated with a longer OS, and this association remained significant in adjusted analysis at both timepoints. Pain and constipation were both independent factors of shorter OS at baseline. Weaker or non-independent associations with survival were seen for role functioning and fatigue at baseline, as well as loss of appetite at baseline and EOT.

## Discussion

The potential associations of inflammatory serum proteins with HRQoL in patients with pancreatic or other periampullary cancers have hitherto remained unexplored. This study, based on real-world data from a prospective, observational cohort, examined these associations over time, and some results merit further attention.

The finding that patients had improved global health and increased appetite, as well as less nausea and vomiting, less constipation and less dyspnoea at three months compared to baseline, i.e., before initiation of chemotherapy, is positive and in line with other studies showing improved quality of life during chemotherapy treatment [9, 10].

Patients with non-central nervous system malignancies often report debilitating problems with memory, attention, and executive functions. These symptoms are referred to as cancer-related cognitive dysfunction (CRCD) or “chemo-brain”. The aetiology behind this condition is not fully understood but believed to be multifactorial, and the subtlety of symptoms makes it difficult to characterise. Neurobiologically, patients with CRCD suffer from hypoactivation of the parietal and prefrontal cortex [38, 39]. In this study, decorin was among the top up-regulated proteins at several timepoints compared to baseline, and patients with higher serum levels of decorin at one month reported a poorer cognitive function at EOT. As emotions are closely linked to cognitive functions such as perception and memory [40], the finding of a significant association between higher levels of serum-decorin at three months and a reduced emotional functioning at EOT further strengthens a link between decorin and the broader term of cognitive functioning. Decorin is a small lysine-rich proteoglycan found in the extracellular matrix, that has been shown to inhibit tumour growth in many cancers, including pancreatic cancer, via interactions with the epidermal growth factor receptor and other members of the ErbB family [41, 42]. Decorin has also been investigated in various neurological conditions. For instance, high levels of decorin have been found in the neurons of the amygdala after traumatic brain injury (TBI) and, hence, decorin is believed to be associated with TBI-associated fear conditioning [43]. Moreover, in mouse models, increased decorin levels in cerebrospinal fluid (CSF) have been shown to be associated with early formation of Aβ amyloidosis and has therefore been proposed as an early biomarker for detection of Alzheimer´s disease [44]. Our findings that increased decorin levels early on during chemotherapy treatment correlated with poorer emotional and/or cognitive functioning later on appear to be well in line with the results from these studies and support its role also in the context of CRCD.

High levels of MCP-1, also called chemokine (C–C motif) ligand 2 (CCL2), at three months were also associated with decreased emotional, but not cognitive, function at EOT. MCP-1 is a glial-derived chemokine which mediates neuroinflammation. MCP-1 levels in serum have been shown to be associated with cognitive decline and decreased memory in older adults, particularly those with Aβ amyloidosis, and to be increased in early-stage Alzheimer’s disease [45–47].

Since CRDC is a complex condition rendering both screening and diagnosis difficult, it would be of value for clinicians to have robust biomarkers to better guide symptom management and patient care. It would therefore be of interest to study the utility of decorin and MCP-1 in this setting, not only in pancreatic but also in other types of cancer.

Various degrees of dyspnoea, or breathlessness, is a common and distressing symptom in patients with advanced cancer, not only in patients with thoracic malignancies [48], and can be attributed to the disease itself, comorbidities and/or the treatment. According to guidelines of the European Society for Medical Oncology (ESMO), patient-reported outcomes are gold standard for assessment of breathlessness, and it is recommended that all cancer patients are screened routinely for this symptom [49]. In this study, high levels of three proteins; GZMB, GZMH, and CD28 at baseline correlated strongly with dyspnoea at EOT. Granzyme B and H are members of the granzyme family of serine proteases [50]. Granzymes are mainly found in cytotoxic granules secreted by cytotoxic T cells and natural killer cells, inducing intracellular death through apoptosis, but increasing evidence suggests that granzymes also activate and amplify immune responses [51]. While granzyme H remains less well studied in terms of respiratory disease, granzyme B has been implicated in e.g. chronic obstructive pulmonary disease [52], and fatal asthma [53]. CD28 is expressed on most cells of the T-lineage, and is an important T cell co-stimulatory receptor that enhances cytokine production, in particular IL-2 [54]. CD28-mediated co-stimulation has been linked to allergic airway inflammation in pre-clinical models [55], but a randomized trial on patients with asthma failed to demonstrate an effect of treatment with the selective costimulatory modulator abatacept, that blocks the interaction with CD28 on T cells [56].

The unveiling of these biomarkers as potential early indicators of dyspnoea/breathlessness in patients with pancreatic cancer should encourage further validatory studies, given the distressing nature of this symptom.

Sleep disturbances are common in cancer patients, with ~ 50% suffering from insomnia, which should be distinguished from cancer-related fatigue. Moreover, sleep disturbances in cancer patients are worsened during chemotherapy treatment [57, 58]. Inflammatory cytokines are often elevated in cancer patients as well as healthy individuals suffering from sleep deprivation [59]. In this study, high levels of four inflammatory proteins; CCL23, CD4, Gal-1, and MMP7, at baseline correlated strongly with insomnia at EOT. The literature provides no clues as to how these proteins may specifically be associated with sleep disturbances. In a recent study by Jensen et al. levels of inflammatory proteins were measured after 3 months treatment in a cohort of mixed cancers. They found an increase in levels of proinflammatory proteins Il-2, Il-6, Il-12, TNF-α, IFN-γ and GM-CSF in patients with impaired sleep quality. All these proteins, apart from GM-CSF, were also included in the panel in our study, but were not found to be associated with insomnia at any timepoint. Taken together, the results from this study do not add sufficient evidence to continue along a specific path towards further validatory study when it comes to potential biomarkers for sleep disturbance.

Optimized management of pain is important to improve cancer patients’ quality of life, and in the present study, pain was an independent factor of shorter survival both at baseline and three months. However, none of the herein investigated proteins correlated strongly with pain. This is in contrast to a previous study exploring the levels of inflammatory proteins in patients with chronic pancreatitis, wherein patients with mild to moderate pain were found to have elevated levels of IL-1β, IL-6, IL-2, tumour necrosis factor α (TNF-α) and MCP-1 and patients with severe pain found to have increased levels of IL-4, IL-8, calcitonin gene receptor peptide (CGRP) and decreased TNF-α [60]. However, while affecting the same organ, and often being pathogenetically interlinked, chronic pancreatitis is a benign, inflammatory disease, whereas pain in pancreatic cancer patients is multifactorial, with both inflammatory and neurogenic components. As pain management is paramount for improved care of cancer patients, future studies on additional patients and biomarker panels are warranted.

Of note, the most significant proteins in terms of correlations with HRQoL factors were not among the top prognostic proteins. Routine laboratory parameters were also more associated with prognosis than with HRQoL factors. This observation further stresses the fact that there is a distinction between disease and illness and, hence, an apparent need to identify biomarkers that align with patient-reported symptoms.

Although additional studies, including more patients, are needed to reach more affirmative conclusions, a strength of this study is the use of multiple longitudinal datapoints, covering up to six months, thus enabling modelling of disease and illness trajectories in individual patients.

## Conclusions

The results from this exploratory study unveil associations of several inflammatory proteins with trajectories of HRQoL during chemotherapy treatment in patients with pancreatic or other periampullary cancer. Early prediction of which patients are likely to be afflicted by a substantial decrease in HRQoL would be an important tool for improved personalized medicine, enabling physicians to adopt a prophylactic approach to symptom management. A diagnosis of pancreatic cancer is a severe crisis for the majority of patients, who often suffer from both major physical and psychological distress. Therefore, while improved therapeutic strategies to combat the disease are highly anticipated, there is also an apparent need for more studies focusing on HRQoL as an outcome.

### Supplementary Information

**Additional file 1.** Correlation coefficients for all serum proteins and all HRQoL factors. Spearman’s rho was calculated for all serum proteins and all HRQoL factors at a) baseline compared to baseline, b) baseline compared to three months, c) baseline compared to EOT, d) one month compared to three months, e) one month compared to EOT, f) three months compared to three months, g) three months compared to EOT and h) EOT compared to EOT, respectively.

**Additional file 2.** Correlations of CEA with Appetite. Spearman’s rho with 95% confidence interval was calculated for CEA with a significant correlation to Appetite. No other significant correlations between routine biomarkers and HRQoL factors were found.

**Additional file 3.** Cox regression analysis of overall survival in relation to levels of routine biomarkers. Hazard ratios with 99.9% confidence intervals for death at all time points in univariable and multivariable analysis, adjusted for treatment intention and performance status at baseline.

**Additional file 4.** Cox regression analysis of overall survival in relation to HRQoL factors. Hazard ratios with 95% confidence intervals for death at all time points in univariable and multivariable analysis, adjusted for sex, performance status (0-1, 2-3) and treatment (adjuvant vs palliative).

## Data Availability

All the data generated in this study are included in the article. Access to all data generated or analysed during this current study will be evaluated according to Swedish legislation and be made available from the corresponding author on reasonable request in allowance with anonymisation and compliance with GDPR standards.
