# Supplementary figures and images for: Trajectories of immune-related serum proteins and quality of life in patients with pancreatic and other periampullary cancer: the CHAMP study
Source: BMC Cancer. 2023 Nov 7;23:1074. doi: 10.1186/s12885-023-11562-2 (PMC10629201; doi:10.1186/s12885-023-11562-2)

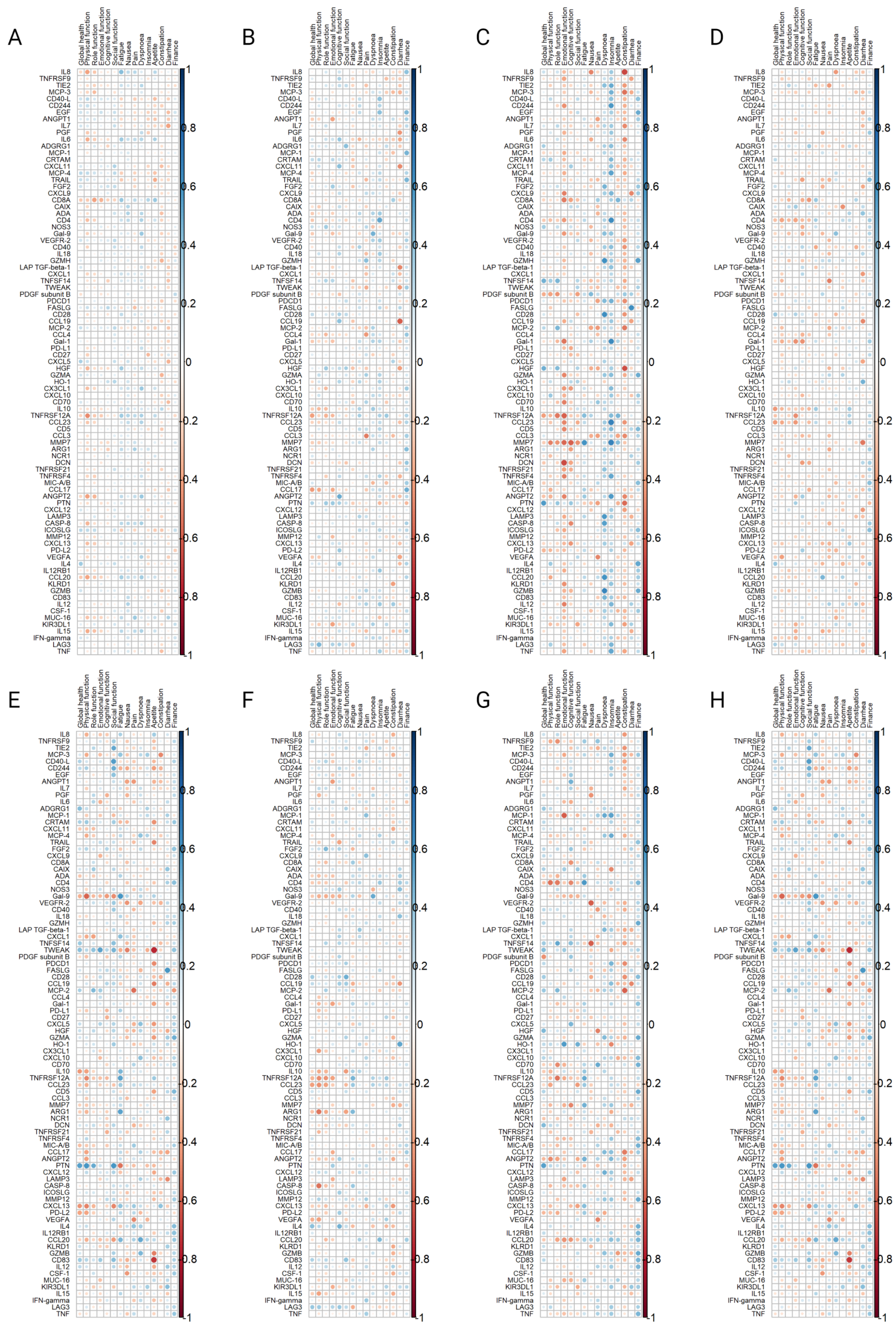

Supplement: Supplementary file 1 — Additional file 1. Correlation coefficients for all serum proteins and all HRQoL factors. Spearman’s rho was calculated for all serum proteins and all HRQoL factors at a) baseline compared to baseline, b) baseline compared to three months, c) baseline compared to EOT, d) one month compared to three months, e) one month compared to EOT, f) three months compared to three months, g) three months compared to EOT and h) EOT compared to EOT, respectively. [file 12885_2023_11562_MOESM1_ESM.pdf]

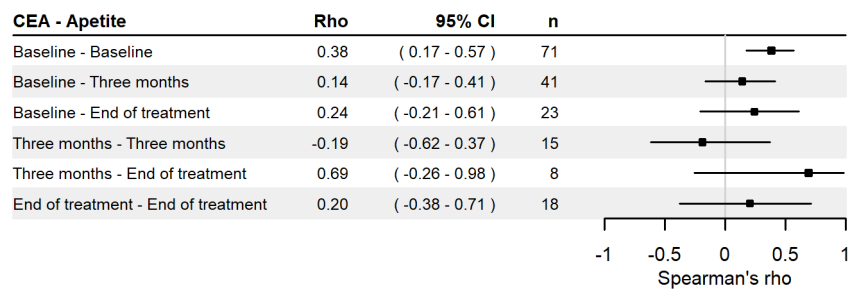

Supplement: Supplementary file 2 — Additional file 2. Correlations of CEA with Appetite. Spearman’s rho with 95% confidence interval was calculated for CEA with a significant correlation to Appetite. No other significant correlations between routine biomarkers and HRQoL factors were found. [file 12885_2023_11562_MOESM2_ESM.pdf]
