# Supplementary material for: Trajectories of immune-related serum proteins and quality of life in patients with pancreatic and other periampullary cancer: the CHAMP study
Source: BMC Cancer. 2023 Nov 7;23:1074. doi: 10.1186/s12885-023-11562-2 (PMC10629201; doi:10.1186/s12885-023-11562-2)

Univariable

Baseline

Parameter	HR	CI	P-value
CRP	1.42	(1.18 - 1.71)	< 0.001
CEA	1.26	(1.13 - 1.40)	< 0.001
CA 19-9	1.17	(1.10 - 1.26)	< 0.001

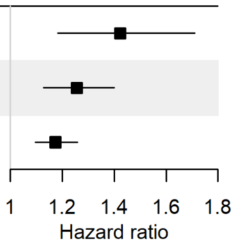

Three months

Parameter	HR	CI	P-value
CEA	1.35	(0.92 - 1.98)	0.130
CA 19-9	1.24	(1.04 - 1.48)	0.017
CRP	1.12	(0.85 - 1.47)	0.434
Albumin	0.17	(0.01 - 2.37)	0.188

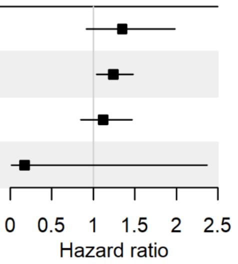

End of treatment

Parameter	HR	CI	P-value
CEA	1.49	(1.25 - 1.77)	< 0.001
CA 19-9	1.18	(1.09 - 1.28)	< 0.001
Albumin	0.12	(0.04 - 0.38)	< 0.001

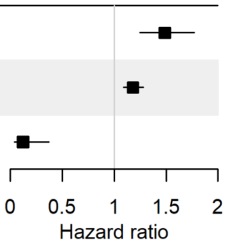

Multivariable

Baseline

Parameter	HR	CI	P-value
CRP	1.41	(1.16 - 1.70)	< 0.001
CEA	1.16	(1.03 - 1.29)	0.011
CA 19-9	1.12	(1.04 - 1.20)	0.002

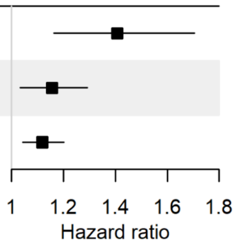

Three months

Parameter	HR	CI	P-value
CEA	1.27	(0.84 - 1.91)	0.263
CA 19-9	1.16	(0.97 - 1.38)	0.115
CRP	1.06	(0.76 - 1.47)	0.739
Albumin	0.61	(0.03 - 12.47)	0.748

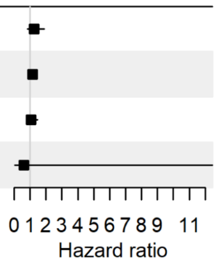

End of treatment

Parameter	HR	CI	P-value
CEA	1.34	(1.11 - 1.61)	0.002
CA 19-9	1.13	(1.04 - 1.23)	0.003
Albumin	0.23	(0.07 - 0.74)	0.014

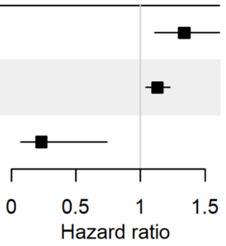

Supplement: Supplementary file 3 — Additional file 3. Cox regression analysis of overall survival in relation to levels of routine biomarkers. Hazard ratios with 99.9% confidence intervals for death at all time points in univariable and multivariable analysis, adjusted for treatment intention and performance status at baseline. [file 12885_2023_11562_MOESM3_ESM.pdf]
