# Supplementary material for: Trajectories of immune-related serum proteins and quality of life in patients with pancreatic and other periampullary cancer: the CHAMP study
Source: BMC Cancer. 2023 Nov 7;23:1074. doi: 10.1186/s12885-023-11562-2 (PMC10629201; doi:10.1186/s12885-023-11562-2)
Supplement: Supplementary file 4 — Additional file 4. Cox regression analysis of overall survival in relation to HRQoL factors. Hazard ratios with 95% confidence intervals for death at all time points in univariable and multivariable analysis, adjusted for sex, performance status (0-1, 2-3) and treatment (adjuvant vs palliative). [file 12885_2023_11562_MOESM4_ESM.docx]

|  | **Hazard ratio for death (95% confidence interval)** | | | | | |
| --- | --- | --- | --- | --- | --- | --- |
|  | **Baseline** | | **3 months** | | **End of treatment** | |
|  | *Univariable* | *Multivariable* | *Univariable* | *Multivariable* | *Univariable* | *Multivariable* |
| Global health status | 0.99 (0.98-1.01) | 1.01 (0.99-1.02) | 1.00 (0.98-1.02) | 1.00 (0.98-1.02) | 1.00 (0.98-1.03) | 1.00 (0.98-1.04) |
| Physical functioning | 0.97 (0.96-0.99)*** | 0.98 (0.96-1.00)* | 0.98 (0.96-1.00)* | 0.98 (0.95-1.00)* | 0.98 (0.95-1.01) | 0.98 (0.94-1.02) |
| Role functioning | 0.99 (0.98-1.00)* | 1.00 (0.99-1.01) | 1.00 (0.98-1.01) | 1.00 (0.98-1.01) | 1.00 (0.99-1.02) | 1.01 (0.99-1.03) |
| Emotional functioning | 0.99 (0.98-1.00) | 1.00 (0.99-1.01) | 1.01 (0.99-1.03) | 1.01 (0.99-1.03) | 1.00 (0.97-1.03) | 1.02 (0.99-1.06 |
| Cognitive functioning | 0.99 (0.98-1.00) | 1.00 (0.98-1.01) | 1.01 (0.99-1.03) | 1.01 (0.99-1.04) | 1.00 (0.98-1.03) | 1.02 (0.98-1.05) |
| Social functioning | 0.996 (0.99-1.01) | 1.01 (1.00-1.02) | 0.99 (0.97-1.00) | 0.00 (0.97-1.01) | 1.00 (0.98-1.02) | 1.00 (0.98-1.03) |
| Fatigue | 1.02 (1.01-1.03)** | 1.01 (1.00-1.02) | 1.01 (0.99-1.02) | 1.00 (0.98-1.02) | 1.01 (0.99-1.02) | 0.00 (0.97-1.02) |
| Nausea and vomiting | 1.01 (1.00-1.03) | 1.00 (0.98-1.02) | 1.00 (0.97-1.03) | 1.05 (1.00-1.10)* | 1.00 (0.95-1.05) | 1.00 (0.95-1.06) |
| Pain | 1.02 (1.01-1.03)*** | 1.02 (1.01-1.02)*** | 1.01 (0.99-1.03) | 1.04 (1.01-1.06)** | 1.00 (0.99-1.02) | 1.00 (0.98-1.02) |
| Dyspnoea | 1.01 (1.00-1.02) | 1.01 (0.99-1.02) | 1.00 (0.98-1.02) | 1.00 (0.98-1.02) | 0.98 (0.96-1.01) | 0.98 (0.95-1.01) |
| Insomnia | 1.00 (0.99-1.01) | 1.00 (0.99-1.01) | 0.99 (0.97-1.00) | 0.99 (0.97-1.01) | 0.99 (0.97-1.01) | 0.98 (0.95-1.00)* |
| Appetite loss | 1.01 (1.01-1.02)*** | 1.01 (1.00-1.02) | 1.01 (1.00-1.02) | 1.01 (1.00-1.02) | 1.02 (1.00-1.04)* | 1.02 (1.00-1.04) |
| Constipation | 1.01 (1.00-1.02)* | 1.01 (1.00-1.02)* | 1.01 (0.99-1.03) | 1.02 (1.00-1.05) | 1.03 (1.00-1.06) | 1.03 (0.99-1.07) |
| Diarrhoea | 1.01 (1.00-1.01) | 0.99 (0.99-1.00) | 1.01 (0.99-1.02) | 1.01 (0.99-1.01) | 1.00 (0.98-1.02) | 1.01 (0.99-1.04) |
| Financial difficulties | 1.00 (0.98-1.01) | 1.00 (0.99-1.02) | 1.00 (0.97-1.02) | 1.01 (0.98-1.03) | 0.94 (0.84-1.05) | N.A. |
